# Levels and durability of neutralizing antibodies against SARS-CoV-2 Omicron and other variants after ChAdOx-1 or BNT162b2 booster in CoronaVac-primed elderly individuals

**DOI:** 10.1016/j.heliyon.2023.e15653

**Published:** 2023-04-20

**Authors:** Nuchjira Takheaw, Chalerm Liwsrisakun, Witida Laopajon, Supansa Pata, Warawut Chaiwong, Juthamas Inchai, Pilaiporn Duangjit, Chaicharn Pothirat, Chaiwat Bumroongkit, Athavudh Deesomchok, Theerakorn Theerakittikul, Atikun Limsukon, Pattraporn Tajarernmuang, Nutchanok Niyatiwatchanchai, Konlawij Trongtrakul, Watchara Kasinrerk

**Affiliations:** a Division of Clinical Immunology, Department of Medical Technology, Faculty of Associated Medical Sciences, Chiang Mai University, Chiang Mai, Thailand; b Biomedical Technology Research Center, National Center for Genetic Engineering and Biotechnology, National Science and Technology Development Agency at the Faculty of Associated Medical Sciences, Chiang Mai University, Chiang Mai, Thailand; c Division of Pulmonary, Critical Care, and Allergy, Department of Internal Medicine, Faculty of Medicine, Chiang Mai University, Chiang Mai, Thailand

**Keywords:** Neutralizing antibody, SARS-CoV-2, Omicron variant, Variants of concern, COVID-19, Booster vaccine, Elderly

## Abstract

The outbreak of the SARS-CoV-2 Omicron variant raised the need for vaccine boosting. We evaluated the efficiency of the third booster vaccine, ChAdOx-1 or BNT162b2, in causing a neutralizing antibody (NAb) response and its durability against the Omicron and other variants in elderly individuals previously vaccinated with 2-dose CoronaVac inactivated vaccine. After receiving 2-dose CoronaVac, only 2.2% of subjects had NAbs against the Omicron variant above the cut-off value. Four weeks after boosting, the number of subjects who had NAb levels above the cut-off values in the ChAdOx-1 and BNT162b2 vaccine boosting groups increased to 41.7% and 54.5%, respectively. However, after 12 and 24 weeks of boosting with any vaccines, NAb levels against the Omicron variant dramatically waned. Twenty-four weeks after boosting, only 2% had high levels of NAbs against the Omicron variant. Compared to other variants, the Omicron variant was less responsive to boosting vaccines. The waning rate of NAb levels for the Omicron variant was much faster than that observed in the Alpha, Beta and Delta variants. To combat the Omicron variant, the fourth booster dose is, therefore, recommended for elderly individuals.

## 1 Introduction

Since the emergence of SARS-CoV-2 in Wuhan, China, in December 2019, this virus has rapidly spread worldwide. To stop the pandemic, various COVID-19 vaccine platforms have been developed, and several vaccines have been authorized for emergency use worldwide [1]. SARS-CoV-2 vaccines are successful in inducing humoral and cellular immunity and reducing SARS-CoV-2 infection, hospitalization, and death [[1], [2], [3], [4], [5]]. The presence of neutralizing antibodies (NAbs) in vaccinated individuals correlates with protection from SARS-CoV-2 infection, and high NAb titres are good predictors for the protection of symptomatic COVID-19 [[6], [7], [8]]. Thus, vaccination is suggested as a promising strategy to prevent COVID-19, particularly to reduce severity and fatality.

In Thailand, CoronaVac, an inactivated vaccine, was the first available vaccine distributed during February–March 2021. Accordingly, health care workers (HCWs) were the first group of the population to receive this vaccine. However, later, mutations in the ancestral virus, especially in the receptor-binding domain (RBD), which is the target site of Nabs, evolved [[9], [10], [11], [12]]. In May 2021, the Delta variant emerged [[13], [14], [15]]. This variant then gradually became a globally dominant variant [9]. The occurrence of the Delta variant caused the breakthrough of COVID-19 among HCWs in Thailand and many countries despite full vaccination with CoronaVac [[14], [15], [16]]. Breakthrough infection of the Delta variant was associated with severe illness, particularly in elderly individuals [16]. The lower susceptibility of the Delta variant to CoronaVac was reported [17,18]. A third booster dose with heterologous vaccine after full 2-dose CoronaVac vaccination was suggested in several countries [[19], [20], [21]]. Since July 2021, in Thailand, a third dose of COVID-19 vaccination either with ChAdOx-1 or BNT162b2 was implemented for HCWs who previously received 2-dose CoronaVac.

Since the emergence of the ancestral Wuhan strain, several VOCs of SARS-CoV-2 have continuously evolved. The latest VOC reported by the World Health Organization (WHO), the Omicron variant, emerged in November 2021. Among the SARS-CoV-2 variants, Omicron was demonstrated to be the most divergent variant. Its mutation sites are widely distributed in many viral structural proteins, including the envelope (E), membrane (M), nucleocapsid (N), and spike (S) proteins [[10], [11], [12]]. Importantly, the Omicron variant has 15 mutated sites located in the RBD and 8 mutations in the NTD of the S1 subunit [11,12]. These mutations increase the infectivity and transmissibility of the Omicron variant. Moreover, the mutations affect the binding of NAbs elicited by the current available COVID-19 vaccines [11,12,22]. Due to the transmissibility and immune evasion ability, the Omicron variant is currently the dominant variant worldwide [10,23]. Recently, the Thai Ministry of Public Health, as well as other countries, implemented a policy of vaccine boosting for not only HCWs but also the whole population to boost the immune response to fight against the Omicron variant.

Elderly individuals are highly vulnerable to a variety of chronic diseases, including cancer, metabolic, cardiovascular, and neurodegenerative diseases, and COVID-19 infection [24,25]. Ageing is a risk factor associated with severe COVID-19 infection as well as poor immune response to vaccination [[26], [27], [28], [29], [30], [31]]. According to vaccine breakthrough infections from Omicron, in Thailand, elderly HCWs who were previously vaccinated with CoronaVac were requested to be boosted with either viral vector or mRNA vaccines. Hence, we raise a concern on whether a third dose of a vaccine can induce the appropriate level of NAbs in elderly individuals, especially for the Omicron variant. In this study, we demonstrated the induction and durability of NAbs against the wild type (WT), Omicron, and other SARS-CoV-2 variants after ChAdOx-1 or BNT162b2 boosting in elderly individuals who received a full dose of CoronaVac. This information is important for designing a suitable vaccination strategy for elderly individuals.

## 2 Participants and methods

### 2.1 Study design and participants

Forty-six HCWs aged ≥60 years and without uncontrolled underlying disease who received 2 doses of a standard regimen of CoronaVac were enrolled in the study at Chiang Mai University Hospital, Chiang Mai, Thailand, from 30th July-1st September 2021. The exclusion criteria and the participants’ demographic data were as presented in our previous study [32]. The demographic data of both groups were not significantly different (Table S1).

This study was approved by the Ethical Committee of the Faculty of Medicine, Chiang Mai University (IRB approval number: MED-2564-08247) and filed under Clinical Trials Registry (Study ID: TCTR20210822002). Before enrollment, written informed consent was obtained from all subjects.

### 2.2 Study procedures

All subjects received two doses of CoronaVac from Sinovac in the previous 4–12 weeks. Subjects were boosted as the third dose with ChAdOx-1(AstraZeneca) or BNT162b2 (BioNTech/Pfizer) based on their own decision. The number, age and sex of the subjects in each boosting group are shown in Table 1. Blood samples were taken immediately prior to booster vaccination and at 4, 12 and 24 weeks after boosting. Plasma was separated and tested for the level of NAb to WT, Omicron, Alpha, Beta and Delta variants.

**Table 1:** Subject information (N = 46).

Information	Boosting with ChAdOx-1	Boosting with BNT162b2
Number of participants	24	22^a^
Average age (years)^b^	70.1 ± 8.3	74.6 ± 9.4
Age range (years)	64–92	60–97
Male	16	10
Female	8	12

a Number of participants is 21 at week 12 and 24.

b Data are exhibited as mean ± SD.

### 2.3 Neutralizing antibody assay

Plasma specimens were determined for NAb against the SARS-CoV-2 WT, Omicron (B.1.1.529), Alpha (B.1.1.7), Beta (B.1.351) and Delta (B.1.617.2) variants by cPass SARS-CoV-2 neutralization antibody detection kit (GenScript, NJ, USA), according to the manufacturer's protocol. Briefly, the kit contains the horseradish peroxidase (HRP) conjugated recombinant SARS-CoV-2 RBD fragment (HRP-RBD) of WT strain and of the VOCs as listed; Omicron (mutation sites at G339D, S371L, S373P, S375F, K417 N, N440K, G446S, S477 N, T478K, E484A, Q493R, G496S, Q498R, N501Y, Y505H), Alpha (Mutation sites at N501Y), Beta (Mutation sites at E484K, K417 N and N501Y) and Delta (Mutation sites at L452R and T478K). The HRP-RBD of each strain was diluted 1:1000 with RBD dilution buffer. The plasma, positive, and negative controls were diluted to 1:9 using sample dilution buffer. The diluted samples and controls were incubated with the HRP-RBD solution at 1:1 ration for 30 min at 37 °C. One hundred microliters of mixtures were added into human angiotensin-converting enzyme 2 (ACE2) coated wells and incubate for 15 min at 37 °C. Unbound proteins were removed by washing for 4 times. The TMB (3,3′,5,5′-Tetramethylbenzidine) substrate solution was added into each well. The plates were incubated for 15 min at room temperature. After the addition of the stop solution, the absorbance was measured at 450 nm using a microtiter plate reader. The % inhibition of NAb was calculated as follows: [1 – (O.D. value of sample/average O.D. value of negative control from the corresponding strain)]×100. According to the manufacturer (GenScript), the 30% inhibition was used as the cut-off, where % inhibition above the cut-off be considered as the NAb for SARS-CoV-2 was detected.

### 2.4 Statistical analysis

The median and interquartile range (IQR) were calculated by Microsoft Excel. All statistical analysis was performed using Graphpad prism v9.2.0 (San Diego, California USA). The Mann-Whitney *U* test was used for comparison the different samples. The Wilcoxon test was used for comparison the paired samples. *P* < 0.05 was considered significant.

## 3 Results

### 3.1 Neutralizing antibody response against the Omicron variant in elderly individuals after vaccine boosting

Forty-six elderly HCWs who received 2-dose CoronaVac were enrolled into the study. Twenty-four and 22 of them were boosted with ChAdOx-1 and BNT162b2, respectively. One of the subjects in the BNT162b2 group died from sepsis 4 weeks after the booster. Therefore, the number of subjects in the BNT162b2 group was 21 at 12 and 24 weeks.

**Fig. 1:**
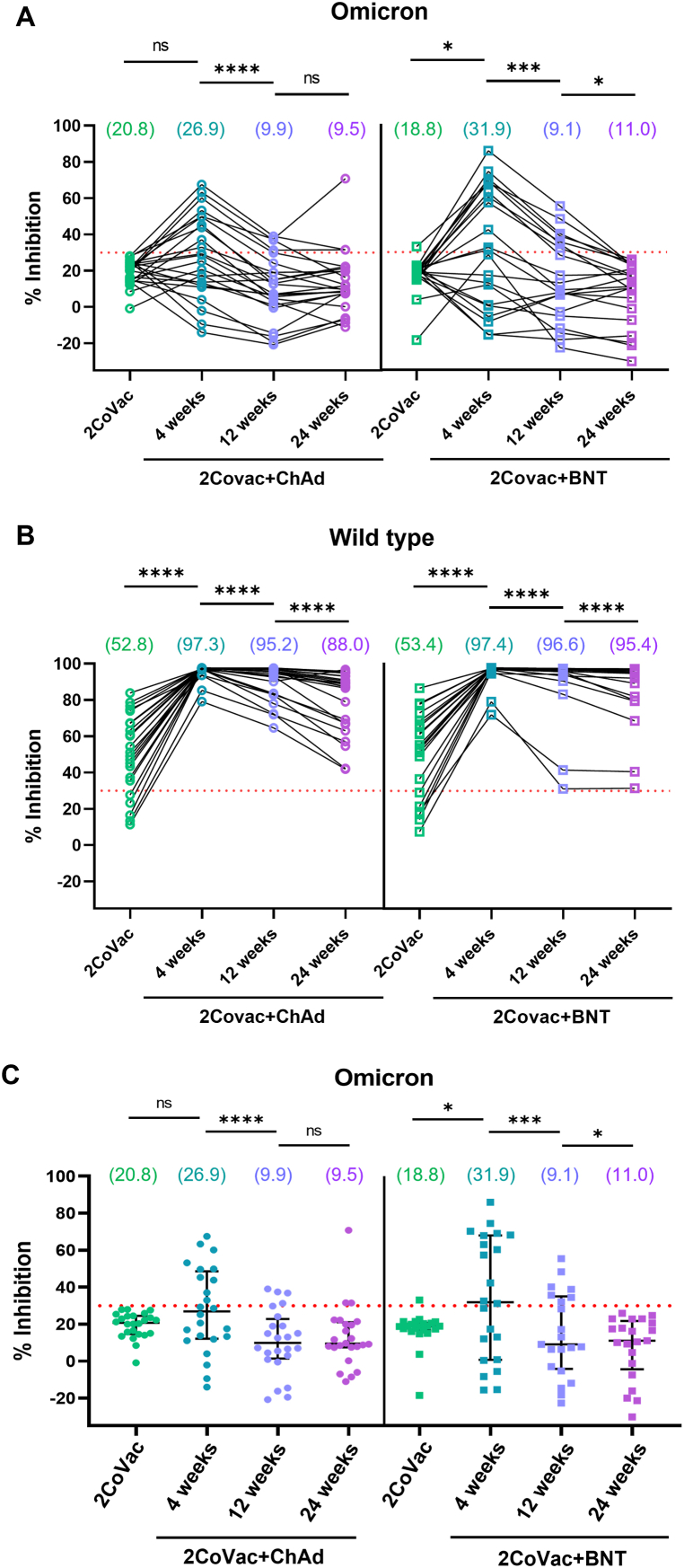
Neutralizing antibody response against the Omicron variant. Blood samples were taken after 2-dose CoronaVac (2CoVac) as a baseline before boosting and after boosting with ChAdOx1 (2CoVac + ChAd) or BNT162b2 (2CoVac + BNT) at 4, 12 and 24 weeks. The % inhibition of NAb response against the Omicron and wild type variants are shown. Dot plots represent each individual, and lines connecting the same person for the NAb response against the Omicron (A) and wild type (B) variants are shown. Scatter dots with the median and interquartile range (IQR) of the NAb response against the Omicron variant at the indicated conditions are shown (C). The numbers in parentheses indicate the median value. The Wilcoxon test was used for comparison of the paired samples, *P < 0.05. ***P < 0.001. ****P < 0.0001. ns = not significant. (The results before and 4 weeks after vaccine boosting have been presented in Immun Ageing. 2022; 19 (1):24. https://doi.org/10.1186/s12979-022-00279-8).

After receiving 2-dose CoronaVac (before boosting), 35 out of 46 (76.1%) subjects had % inhibition of NAbs against the WT strain above the 30% cut-off for NAb detection. However, only 1 out of 46 (2.2%) subjects had % inhibition of NAbs against the Omicron variant above the cut-off. Four weeks after boosting, the number of subjects who had % inhibition of NAbs against the Omicron variant above the 30% cut-off in the ChAdOx-1 and BNT162b2 groups were 41.7% and 54.5%, respectively (Fig. 1A). However, at 12 and 24 weeks, a decrement in NAb levels was observed for both boosting vaccines (Fig. 1A). The decremental rate of NAb levels against Omicron was higher than that against the WT strain (Fig. 1A vs. Fig. 1B). The levels of NAbs against the Omicron after boosting with ChAdOx1 or BNT162b2 were (median) 26.9% (IQR 12.9–46.4) vs. 31.9% (IQR 3.6–66.7) at 4 weeks, 9.9% (IQR 2.5–20.3) vs. 9.1% (IQR -0.2–34.4) at 12 weeks and 9.5% (IQR 7.5–20.3) vs. 11.0% (IQR -0.3–20.6) at 24 weeks (Fig. 1C). Twenty-four weeks after boosting, subjects who had NAb levels above the NAb detection cut-off for the WT and Omicron variants were 100% and 6.7%, respectively (Tables S2 and S3). It is worth mentioning that the % inhibition of NAbs against Omicron in 3 out of 45 subjects who had a % of inhibition of NAbs above the 30% cut-off was 31.5%, 31.6% and 70.8%, indicating that only 1 subject had evident NAbs (Table S2).

**Fig. 2:**
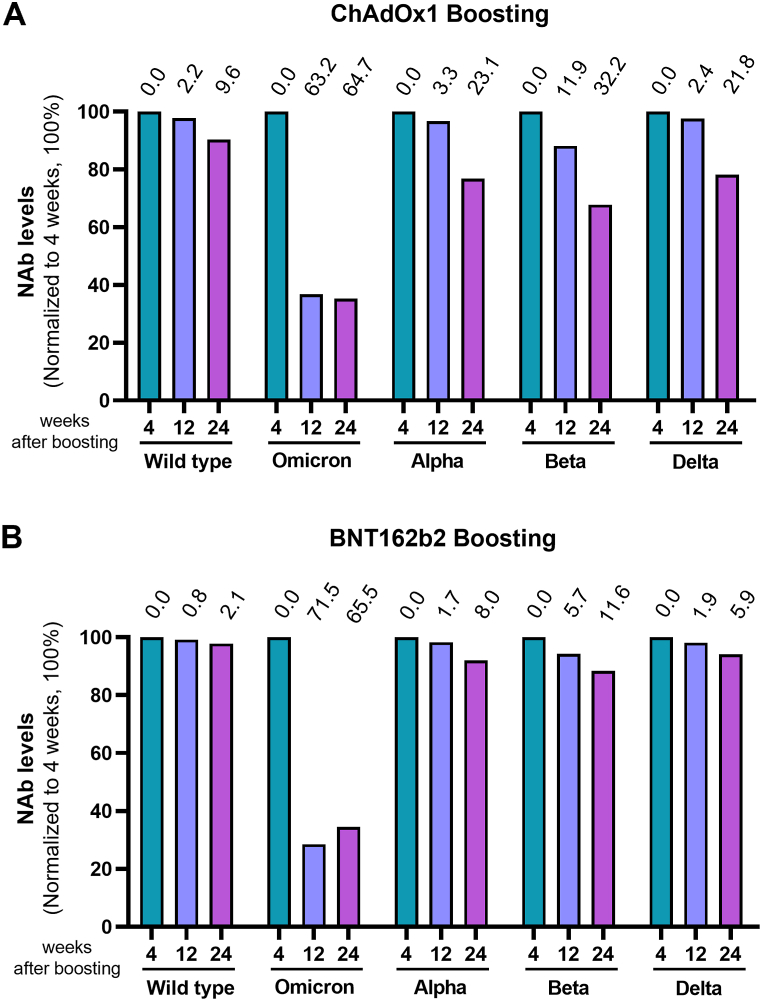
Decrease in neutralizing antibody levels against SARS-CoV-2 variants. The % inhibition (median) of NAb response against the indicated variants at weeks 12 and 24 after ChAdOx1 (A) and BNT162b2 (B) boosting were normalized to the % inhibition (median) of NAb response at week 4 as 100%. The numbers above the bar graph indicate the % of the decrement in NAb levels at the indicated weeks compared to week 4.

The NAb responses against WT and Omicron after vaccine boosting at 4, 12 and 24 weeks were compared. The NAb levels at 12 and 24 weeks were normalized to the NAb level at 4 weeks as 100% (Fig. 2). After boosting with ChAdOx1, the % of the decrement in NAb levels against WT vs. Omicron at 12 and 24 weeks from that at 4 weeks was 2.2% vs. 63.2% and 9.6% vs. 64.7%, respectively (Fig. 2A). Similarly, after boosting with BNT162b2, the % of the decrement in NAb levels against the WT vs. Omicron strain at 12 and 24 weeks from 4 weeks was 0.8% vs. 71.5% and 2.1% vs. 65.5%, respectively (Fig. 2B). The results indicated that after 12 and 24 weeks of boosting with any vaccines, the levels of NAbs against Omicron were dramatically reduced.

### 3.2 Neutralizing antibody response against the Alpha, Beta and Delta variants in elderly individuals after vaccine boosting

**Fig. 3:**
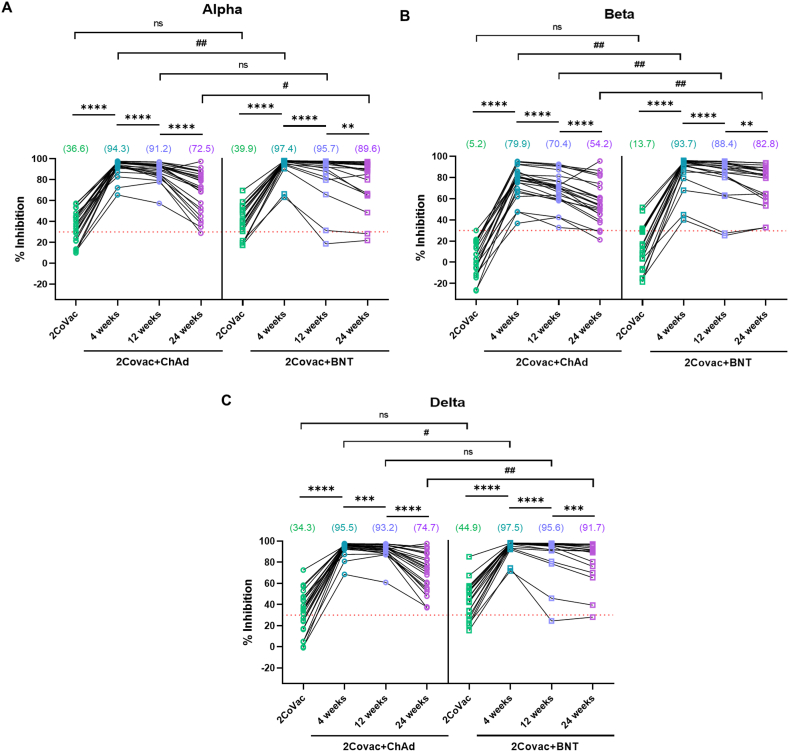
Neutralizing antibody response against the Alpha, Beta and Delta variants. Blood samples were taken after 2-dose CoronaVac (2CoVac) as a baseline before boosting and after boosting with ChAdOx1 (2CoVac + ChAd) or BNT162b2 (2CoVac + BNT) at 4, 12 and 24 weeks. The % inhibition of the NAb response against Alpha (A), Beta (B) and Delta (C) variants is shown. Dot plots represent an individual, and lines connecting the same person are shown. The numbers in parentheses indicate the median value. The Wilcoxon test was used for comparison of the paired samples, **P < 0.01. ***P < 0.001. ****P < 0.0001. The Mann–Whitney *U* test was used for comparison of the different samples, ^#^*P* < 0.05. ^##^*P* < 0.01. ns = not significant. (The results before and 4 weeks after vaccine boosting have been presented in Immun Ageing. 2022; 19 (1):24. https://doi.org/10.1186/s12979-022-00279-8).

For the Alpha variant, as shown in Fig. 3A, the % inhibition of NAbs against the Alpha variant before and at 4, 12, and 24 weeks after ChAdOx-1 boosting were (median) 36.6% (IQR 23.4–44.6), 94.3% (IQR 92.4–96.6), 91.2% (IQR 85.0–93.9) and 72.5% (IQR 52.0–83.8), respectively. For the BNT162b2 boosting group, the % inhibition of NAbs were (median) 39.9% (IQR 30.8–51.0), 97.4% (IQR 94.8–97.9), 95.7% (IQR 87.6–96.8) and 89.6% (IQR 66.5–94.6) in the same sequence (Fig. 3A). After boosting with any vaccines, the levels of NAbs declined with time (Fig. 2).

For the Beta variant, the % inhibition of NAbs before and at 4, 12, and 24 weeks after ChAdOx-1 vs. BNT162b2 boosting were 5.2% (IQR -5.4–18.7) vs. 13.7% (IQR 3.6–30.5), 79.9% (IQR 68.9–84.7) vs. 93.7% (IQR 88.5–95.4), 70.4% (IQR 60.6–77.6) vs. 88.4% (IQR 82.3–93.4) and 54.2% (IQR 45.2–71.3) vs. 82.8% (IQR 64.5–86.8), respectively (Fig. 3B). The level of NAbs against Beta was significantly higher in the BNT162b2 boosting group than in the ChAdOx1 boosting group at all time points (Fig. 3B). After boosting with any vaccines, the levels of NAbs against the Beta variant also declined over time (Fig. 2).

For the Delta variant, in the ChAdOx1 boosting group, the % inhibition of NAbs before boosting was (median) 34.3% (IQR 17.1–44.7) (Fig. 3C). After boosting with ChAdOx1, the % inhibition of NAbs were (median) 95.5% (IQR 93.8–96.9), 93.2% (IQR 90.4–95.2) and 74.7% (IQR 58.6–88.2) at 4, 12 and 24 weeks, respectively (Fig. 3C). For the BNT162b2 boosting group, the % inhibition of NAbs was (median) 44.9% (IQR 30.0–53.3) before boosting and 97.5% (IQR 95.0–97.9), 95.6% (IQR 91.5–97.4) and 91.7% (IQR 80.4–96.6) after boosting at 4, 12 and 24 weeks, respectively (Fig. 3C). As predicted, after boosting with any vaccines, the levels of NAbs declined from 4, 12, and 24 weeks (Fig. 2).

The rates of NAbs against the Omicron variant waned much faster than those observed for the Alpha, Beta and Delta variants (Fig. 2).

## 4 Discussion

In Thailand, elderly HCWs were the first group to receive 2-dose CoronaVac, which was the first available vaccine in Thailand. CoronaVac, a chemosynthetic inactivated vaccine, has been reported to be safe and well tolerated in elderly individuals [33,34]. Induction of a sufficient NAb titre against COVID-19 illness by this vaccine type has been reported [34,35]. However, the data were published before the existence of the Delta variant. After the emergence of the Delta variant, however, breakthrough infection from this variant, in association with severity and hospitalization, among HCWs despite full CoronaVac vaccination occurred in Thailand and many countries [12,15,16]. Several reports demonstrated less sensitivity of the Delta variant to the CoronaVac vaccine [17,18]. The third booster vaccine, ChAdOx-1 or BNT162b2, was administered to elderly HCWs in Thailand to combat Delta variant infection.

Later, the Omicron variant developed and rapidly became the dominant globally circulating strain. Several reports have demonstrated the resistance of the Omicron strain to the immunity induced by COVID-19 vaccines [[36], [37], [38]]. Two-dose primary vaccines may not be sufficient to protect against Omicron infection. Hence, vaccination with a third dose has been recommended in many countries worldwide. In Thailand, as mentioned, elderly HCWs previously immunized with CoronaVac received the third dose of either viral vector vaccine ChAdOx-1 or mRNA vaccine BNT162b2. Nevertheless, we wondered whether these boosting regimens could induce a NAb response against the Omicron variant and how long NAbs persisted after vaccine boosting.

As expected, the levels of NAbs against the parental Wuhan WT strain robustly increased at 4 weeks after either ChAdOx1 or BNT162b2 boosting and gradually decreased at 12 and 24 weeks. This phenomenon was the same as previous reports indicating the generation of immunological memory by the inactivated vaccine [21,39]. Compared to the Omicron variant, after 2-dose CoronaVac (before boosting), majority of the tested subjects had NAbs against the Omicron variant below the cut-off value. Omicron has a large number of mutations, particularly at the S protein, which cause evasion of this variant to vaccine-induced antibodies [21,39,40]. Our results confirm the ineffective protective effect of CoronaVac against Omicron infection [21,39]. Four weeks after boosting either with ChAdOx1 or BNT162b2, there were variable NAb responses. Most participants, although they had increased levels of NAb response to the booster vaccine compared to the baseline before receiving a booster, the median NAb level was marginal to the cut-off of 30%, particularly in the ChAdOx-1 group. Moreover, some individuals in both groups had decreased levels of NAbs after 4 weeks of the booster in comparison with pre-booster levels. This phenomenon was observed only with the Omicron variant but not with the other tested VOCs. Unresponsiveness to the booster vaccine in the induction of NAbs against Omicron observed in the present report was in accordance and not in accordance with previous reports [21,[39], [40], [41], [42], [43]]. The primary vaccine, booster vaccine used, and subjects’ ages in this study, however, were different from those in previous reports. To the best of our knowledge, there have been no previous reports on the efficiency of vaccine boosting following inactivated vaccination in elderly individuals.

The duration of the NAb response to Omicron was investigated. A robust decline in NAb levels against Omicron was observed at 12 and 24 weeks. At 24 weeks after vaccine boosting with ChAdOx1 or BNT162b2, only one subject had a NAb level against the Omicron variant higher than the cut-off value. This subject, however, showed an uncommon pattern. The NAb response at 24 weeks was higher than that at 12 weeks after vaccine boosting. We speculated that this subject might be asymptomatically infected with Omicron. The decrement over time in the NAb response against other tested variants, i.e., Alpha, Beta, and Delta, was also observed. However, the rates at which the response waned were much slower than those observed with the Omicron variant. The Omicron variant is the most mutated strain among the VOCs. Hence, the Omicron strain can better evade the NAbs induced by vaccination compared with the other three VOCs.

There were some limitations of our study. The number of subjects used in our study was a small sample size. This study was done in HCWs of Chiang Mai University Hospital, Chiang Mai, Thailand. Thus, a larger sample size in different careers and geographical areas are required for confirming these results.

## 5 Conclusion

In the present study, we compared the immunogenicity of the booster vaccines to the Omicron and other VOCs in elderly individuals who previously received CoronaVac. We demonstrated that the Omicron variant was less responsive to vaccines with rapid NAb waning, despite boosting with a third dose with either viral vector or mRNA vaccines. As the Omicron variant becomes the dominant strain of SARS-CoV-2 in global circulation, this creates concern for elderly individuals who receive inactivated vaccines. The third dose of heterologous vaccine may not be enough to raise NAb levels to the protective level needed for the Omicron strain. A fourth dose, perhaps, needs to be considered. However, it is important to note that CoronaVac has been shown to elicit T-cell responses, which may be involved in protection against infection, at least reducing severity and death [36]. Our findings might have immediate implications for countries that previously used a 2-dose regimen of CoronaVac for elderly individuals.

## Author contribution statement

Chalerm Liwsrisakun; Watchara Kasinrerk: Conceived and designed the experiments; Analyzed and interpreted the data; Wrote the paper.

Nuchjira Takheaw; Witida Laopajon; Supansa Pata: Performed the experiments; Analyzed and interpreted the data; Wrote the paper.

Warawut Chaiwong: Analyzed and interpreted the data; Wrote the paper.

Chaiwat Bumroongkit: Performed the experiments.

Juthamas Inchai; Pilaiporn Duangjit; Chaicharn Pothirat; Athavudh Deesomchok; Theerakorn Theerakittikul; Atikun Limsukon; Pattraporn Tajarernmuang; Nutchanok Niyatiwatchanchai; Konlawij Trongtrakul: Contributed reagents, materials, analysis tools or data.

## Data availability statement

Data included in article/supp. material/referenced in article.

## Declaration of interest’s statement

The authors declare no competing interests.

## References

[1] Nagy A., Alhatlani B. (2021). An overview of current COVID-19 vaccine platforms. Comput. Struct. Biotechnol. J..

[2] Sheikh A., Robertson C., Taylor B. (2021). BNT162b2 and ChAdOx1 nCoV-19 vaccine effectiveness against death from the delta variant. N. Engl. J. Med..

[3] Baden L.R. (2021). Efficacy and safety of the mRNA-1273 SARS-CoV-2 vaccine. N. Engl. J. Med..

[4] Sadoff J. (2021). Safety and efficacy of single-dose Ad26.COV2.S vaccine against covid-19. N. Engl. J. Med..

[5] Tregoning J.S. (2021). Progress of the COVID-19 vaccine effort: viruses, vaccines and variants versus efficacy, effectiveness and escape. Nat. Rev. Immunol..

[6] Khoury D.S. (2021). Neutralizing antibody levels are highly predictive of immune protection from symptomatic SARS-CoV-2 infection. Nat. Med..

[7] Addetia A. (2020). Neutralizing antibodies correlate with protection from SARS-CoV-2 in humans during a fishery vessel outbreak with a high attack rate. J. Clin. Microbiol..

[8] McMahan K. (2021). Correlates of protection against SARS-CoV-2 in rhesus macaques. Nature.

[9] Khateeb J., Li Y., Zhang H. (2021). Emerging SARS-CoV-2 variants of concern and potential intervention approaches. Crit. Care.

[10] Tian D. (2022). The emergence and epidemic characteristics of the highly mutated SARS-CoV-2 Omicron variant. J. Med. Virol..

[11] Dejnirattisai W. (2022). SARS-CoV-2 Omicron-B.1.1.529 leads to widespread escape from neutralizing antibody responses. Cell.

[12] Cui Z. (2022). Structural and functional characterizations of infectivity and immune evasion of SARS-CoV-2 Omicron. Cell.

[13] Novelli G., Colona V.L., Pandolfi P.P. (2021). A focus on the spread of the delta variant of SARS-CoV-2 in India. Indian J. Med. Res..

[14] Waldman S.E. (2022). Secondary cases of delta variant coronavirus disease 2019 among vaccinated healthcare workers with breakthrough infections is rare. Clin. Infect. Dis..

[15] Chau N.V.V. (2021). An observational study of breakthrough SARS-CoV-2 Delta variant infections among vaccinated healthcare workers in Vietnam. EClinical Med..

[16] Bosch W. (2022). Coronavirus disease 2019 vaccine-breakthrough infections requiring hospitalization in mayo clinic Florida through august 2021. Clin. Infect. Dis..

[17] Melo-Gonzalez F. (2021). Recognition of variants of concern by antibodies and T cells induced by a SARS-CoV-2 inactivated vaccine. Front. Immunol..

[18] Vacharathit V. (2021). CoronaVac induces lower neutralising activity against variants of concern than natural infection. Lancet Infect. Dis..

[19] Souza W.M. (2021). Neutralisation of SARS-CoV-2 lineage P.1 by antibodies elicited through natural SARS-CoV-2 infection or vaccination with an inactivated SARS-CoV-2 vaccine: an immunological study. Lancet Microb..

[20] Jantarabenjakul W. (2022). Short-term immune response after inactivated SARS-CoV-2 (CoronaVac(R), Sinovac) and ChAdOx1 nCoV-19 (Vaxzevria(R), Oxford-AstraZeneca) vaccinations in health care workers. Asian Pac. J. Allergy Immunol..

[21] Perez-Then E. (2022). Neutralizing antibodies against the SARS-CoV-2 Delta and Omicron variants following heterologous CoronaVac plus BNT162b2 booster vaccination. Nat. Med..

[22] Shah M., Woo H.G. (2021). Omicron: a heavily mutated SARS-CoV-2 variant exhibits stronger binding to ACE2 and potently escapes approved COVID-19 therapeutic antibodies. Front. Immunol..

[23] Sharma V. (2022). Emerging evidence on Omicron (B.1.1.529) SARS-CoV-2 variant. J. Med. Virol..

[24] Esteve A. (2020). National age and coresidence patterns shape COVID-19 vulnerability. Proc. Natl. Acad. Sci. U. S. A..

[25] Perrotta F. (2020). COVID-19 and the elderly: insights into pathogenesis and clinical decision-making. Aging Clin. Exp. Res..

[26] Gustafson C.E. (2020). Influence of immune aging on vaccine responses. J. Allergy Clin. Immunol..

[27] McElhaney J.E. (2020). The immune response to influenza in older humans: beyond immune senescence. Immun. Ageing.

[28] Fang X. (2020). Epidemiological, comorbidity factors with severity and prognosis of COVID-19: a systematic review and meta-analysis. Aging (Albany NY).

[29] Pijls B.G. (2021). Demographic risk factors for COVID-19 infection, severity, ICU admission and death: a meta-analysis of 59 studies. BMJ Open.

[30] Cardinali D.P. (2020). Elderly as a high-risk group during COVID-19 pandemic: effect of circadian misalignment, sleep dysregulation and melatonin administration. Sleep Vigil.

[31] Ciabattini A. (2018). Vaccination in the elderly: the challenge of immune changes with aging. Semin. Immunol..

[32] Liwsrisakun C. (2022). Neutralizing antibody and T cell responses against SARS-CoV-2 variants of concern following ChAdOx-1 or BNT162b2 boosting in the elderly previously immunized with CoronaVac vaccine. Immun. Ageing.

[33] Wu Z. (2021). Safety, tolerability, and immunogenicity of an inactivated SARS-CoV-2 vaccine (CoronaVac) in healthy adults aged 60 years and older: a randomised, double-blind, placebo-controlled, phase 1/2 clinical trial. Lancet Infect. Dis..

[34] Tanriover M.D. (2021). Efficacy and safety of an inactivated whole-virion SARS-CoV-2 vaccine (CoronaVac): interim results of a double-blind, randomised, placebo-controlled, phase 3 trial in Turkey. Lancet.

[35] Ranzani O.T. (2021). Effectiveness of the CoronaVac vaccine in older adults during a gamma variant associated epidemic of covid-19 in Brazil: test negative case-control study. BMJ.

[36] Mok C.K.P. (2021).

[37] Zhang L. (2022). The significant immune escape of pseudotyped SARS-CoV-2 variant Omicron. Emerg. Microb. Infect..

[38] Muik A. (2022). Neutralization of SARS-CoV-2 Omicron by BNT162b2 mRNA vaccine-elicited human sera. Science.

[39] Cheng S.M.S. (2022). Neutralizing antibodies against the SARS-CoV-2 Omicron variant BA.1 following homologous and heterologous CoronaVac or BNT162b2 vaccination. Nat. Med..

[40] Ai J. (2022). Omicron variant showed lower neutralizing sensitivity than other SARS-CoV-2 variants to immune sera elicited by vaccines after boost. Emerg. Microb. Infect..

[41] Garcia-Beltran W.F. (2022). mRNA-based COVID-19 vaccine boosters induce neutralizing immunity against SARS-CoV-2 Omicron variant. Cell.

[42] Cele S. (2022). Omicron extensively but incompletely escapes Pfizer BNT162b2 neutralization. Nature.

[43] Lu L. (2022). Neutralization of Severe Acute Respiratory Syndrome Coronavirus 2 Omicron variant by sera from BNT162b2 or CoronaVac vaccine recipients. Clin. Infect. Dis..

