# Supplementary material for: Levels and durability of neutralizing antibodies against SARS-CoV-2 Omicron and other variants after ChAdOx-1 or BNT162b2 booster in CoronaVac-primed elderly individuals
Source: Heliyon. 2023 Apr 20;9(4):e15653. doi: 10.1016/j.heliyon.2023.e15653 (PMC10116116; doi:10.1016/j.heliyon.2023.e15653)
Supplement: Multimedia component 1 [file mmc1.docx]

**Table S1: Demographic data of the study population (N=46)**

| **Demographic data** | **ChAdOx-1 (N=24)** | **BNT162b2 (N=22)** | **p value** |
| --- | --- | --- | --- |
| Age (years) | 70.1 ± 8.3  (min-max, 64-92) | 74.6 ± 9.4  (min-max, 60-97) | 0.093 |
| BMI (kg/m^2^) | 23.9 ± 2.8 | 23.3 ± 4.3 | 0.547 |
| Sex (male) | 16 (66.7) | 10 (45.5) | 0.234 |
| Smoking status |  |  | 1.000 |
| Nonsmoker | 22 (91.7) | 20 (90.9) |  |
| Ex-smoker | 2 (8.3) | 2 (9.1) |  |
| Underlying diseases |  |  | 0.505 |
| Cardiovascular | 11 (45.8) | 13 (59.1) |  |
| Respiratory | 4 (16.7) | 0 (0.0) |  |
| Metabolic | 1 (4.2) | 2 (9.1) |  |
| Neuromuscular | 1 (4.2) | 2 (9.1) |  |
| Cardiovascular and respiratory | 1 (4.2) | 1 (4.5) |  |
| Cardiovascular and metabolic | 2 (8.3) | 1 (4.5) |  |
| Respiratory and metabolic | 1 (4.2) | 0 (0.0) |  |
| Respiratory and gastrointestinal | 1 (4.2) | 0 (0.0) |  |
| None | 2 (8.3) | 3 (13.6) |  |

Data are presented as the mean ± SD or N (%)

This demographic data is the same as those published in Immun Ageing. 2022;19(1):24. doi: 10.1186/s12979-022-00279-8.

**Table S2. % Inhibition of neutralizing** **antibody against SARS-CoV-2 wild type, Omicron, Alpha, Beta and Delta variants after ChAdOx1 boosting**

| **No.** | **Wild type** | | | | **Omicron** | | | | **Alpha** | | | | **Beta** | | | | **Delta** | | | |
| --- | --- | --- | --- | --- | --- | --- | --- | --- | --- | --- | --- | --- | --- | --- | --- | --- | --- | --- | --- | --- |
|  | **B** | **4w** | **12w** | **24w** | **B** | **4w** | **12w** | **24w** | **B** | **4w** | **12w** | **24w** | **B** | **4w** | **12w** | **24w** | **B** | **4w** | **12w** | **24w** |
| 1 | 78.7 | 97.5 | 96.8 | 91.1 | -0.9 | 17.5 | -0.5 | 7.5 | 56.6 | 96.7 | 93.9 | 78.7 | 29.9 | 77.3 | 70.9 | 53.9 | 57.2 | 96.0 | 94.3 | 77.9 |
| 2 | 16.5 | 97.2 | 93.2 | 66.6 | 25.9 | -2.1 | -14.5 | 7.9 | 9.9 | 93.9 | 83.2 | 44.8 | -14.6 | 69.4 | 59.6 | 37.4 | 4.9 | 96.8 | 91.6 | 52.2 |
| 3 | 11.3 | 79.1 | 64.6 | 41.9 | 16.5 | 13.7 | 15.0 | 22.3 | 11.4 | 65.4 | 57.3 | 34.7 | -26.2 | 47.7 | 32.8 | 29.9 | -0.1 | 68.6 | 60.9 | 37.7 |
| 4 | 54.4 | 97.7 | 96.9 | 93.2 | 12.2 | 37.0 | 4.5 | 0.2 | 35.5 | 97.0 | 94.8 | 87.8 | 19.2 | 94.7 | 91.2 | 81.5 | 34.5 | 97.5 | 96.4 | 89.6 |
| 5 | 67.2 | 97.4 | 96.9 | 93.1 | 23.5 | 29.3 | 13.0 | 19.9 | 40.5 | 92.6 | 92.0 | 83.2 | 19.9 | 77.8 | 72.4 | 57.6 | 46.3 | 96.2 | 94.8 | 83.2 |
| 6 | 35.5 | 97.4 | 97.3 | 94.9 | 24.3 | 32.9 | 19.0 | 19.5 | 21.3 | 95.8 | 93.3 | 85.4 | -12.7 | 67.2 | 58.1 | 50.3 | 16.5 | 97.5 | 97.3 | 94.5 |
| 7 | 74.8 | 93.6 | 72.4 | 42.2 | 8.5 | 20.6 | 5.5 | 8.8 | 48.1 | 82.7 | 79.1 | 28.7 | -2.0 | 47.4 | 42.3 | 21.2 | 47.4 | 87.4 | 87.8 | 37.0 |
| 8 | 37.3 | 96.7 | 82.8 | 54.6 | 22.9 | 11.8 | 6.6 | 8.5 | 21.5 | 92.3 | 84.3 | 40.7 | -5.9 | 81.6 | 66.1 | 45.2 | 16.7 | 94.0 | 90.5 | 47.8 |
| 9 | 49.0 | 97.5 | 97.6 | 95.1 | 21.2 | 63.3 | 36.8 | 16.3 | 30.7 | 97.6 | 96.6 | 87.7 | 18.6 | 95.2 | 92.5 | 83.2 | 24.8 | 97.3 | 97.5 | 92.8 |
| 10 | 23.2 | 97.0 | 83.6 | 67.2 | 13.4 | 45.3 | 12.5 | 9.7 | 12.4 | 93.8 | 85.3 | 49.9 | -27.0 | 75.7 | 60.9 | 39.6 | -0.9 | 93.2 | 88.3 | 54.2 |
| 11 | 13.3 | 85.3 | 71.9 | 57.1 | 14.5 | 11.0 | 7.0 | 11.6 | 13.6 | 72.2 | 77.9 | 38.2 | -11.0 | 36.7 | 42.4 | 28.5 | 4.9 | 80.9 | 87.1 | 59.6 |
| 12 | 76.7 | 97.4 | 92.6 | 89.5 | 14.0 | -14.0 | -20.8 | -8.6 | 53.2 | 95.8 | 92.6 | 79.4 | -5.2 | 84.3 | 76.4 | 59.7 | 17.2 | 94.4 | 91.6 | 73.0 |
| 13 | 60.9 | 97.7 | 97.6 | 94.9 | 20.3 | 53.2 | 29.8 | 22.2 | 43.9 | 97.7 | 97.0 | 88.4 | 19.8 | 92.3 | 87.3 | 70.3 | 44.2 | 97.1 | 96.7 | 88.0 |
| 14 | 74.0 | 97.3 | 95.8 | 87.2 | 15.7 | 25.2 | 3.0 | -11.1 | 53.5 | 96.6 | 93.0 | 68.9 | 13.2 | 75.0 | 69.7 | 53.2 | 58.4 | 96.6 | 95.0 | 75.2 |
| 15 | 45.0 | 97.4 | 95.6 | 87.4 | 28.0 | 43.8 | 14.9 | 21.4 | 28.9 | 94.5 | 89.2 | 71.7 | -0.1 | 65.8 | 59.2 | 48.6 | 36.7 | 96.7 | 94.0 | 77.6 |
| 16 | 67.1 | 96.9 | 90.1 | 96.8 | 27.6 | 49.9 | 37.4 | 70.8 | 45.6 | 92.4 | 88.0 | 97.4 | 20.8 | 81.2 | 72.3 | 95.6 | 36.0 | 95.2 | 92.5 | 97.6 |
| 17 | 83.9 | 97.0 | 93.5 | 86.5 | 22.1 | 49.7 | 24.1 | 12.2 | 57.5 | 93.0 | 86.4 | 71.3 | 7.4 | 81.6 | 69.9 | 61.1 | 72.6 | 95.6 | 92.7 | 81.8 |
| 18 | 27.6 | 97.1 | 78.0 | 63.1 | 15.0 | 4.0 | -19.5 | -6.1 | 24.0 | 94.1 | 81.4 | 52.7 | 2.8 | 85.9 | 67.7 | 54.6 | 24.0 | 94.6 | 88.1 | 55.7 |
| 19 | 43.3 | 97.5 | 95.6 | 89.5 | 27.9 | 67.5 | 39.0 | 31.5 | 33.3 | 96.9 | 93.6 | 80.7 | 18.1 | 95.0 | 91.4 | 86.6 | 38.9 | 97.0 | 96.0 | 88.5 |
| 20 | 63.3 | 97.7 | 97.6 | 95.8 | 20.1 | 60.1 | 31.3 | 31.6 | 44.0 | 97.5 | 96.8 | 91.6 | 14.9 | 92.6 | 90.6 | 83.4 | 53.0 | 97.7 | 97.4 | 94.3 |
| 21 | 60.2 | 97.2 | 96.0 | 90.4 | 20.0 | 13.3 | 0.9 | 7.4 | 44.2 | 95.6 | 93.9 | 81.7 | 7.4 | 82.3 | 81.2 | 74.3 | 27.2 | 95.4 | 93.7 | 74.3 |
| 22 | 51.1 | 93.7 | 83.1 | 69.2 | 25.4 | -9.5 | -16.2 | -7.0 | 37.7 | 87.2 | 85.3 | 57.7 | 21.5 | 79.5 | 76.2 | 60.9 | 43.6 | 92.2 | 89.9 | 61.1 |
| 23 | 67.3 | 97.7 | 97.0 | 79.0 | 22.2 | 28.6 | 7.2 | 8.1 | 42.1 | 96.6 | 94.2 | 68.3 | -4.8 | 80.3 | 71.2 | 44.9 | 31.3 | 95.3 | 93.8 | 70.3 |
| 24 | 47.1 | 96.6 | 94.7 | 88.6 | 24.5 | 16.9 | 18.9 | 9.2 | 30.5 | 90.7 | 90.4 | 73.3 | 3.1 | 62.0 | 63.4 | 51.1 | 34.0 | 93.0 | 91.7 | 68.0 |

B = 2 dose-CoronaVac (before boosting); 4w, 12w, 24w = 4 weeks, 12 weeks and 24 weeks after ChAdOx1 boosting.

Red letter indicates the subject may be asymptomatically infected with SARS-CoV-2.

**Table S3. % Inhibition of neutralizing antibody against SARS-CoV-2 wild type, Omicron, Alpha, Beta and Delta variants after BNT162b2 boosting**

| **No.** | **Wild type** | | | | **Omicron** | | | | **Alpha** | | | | **Beta** | | | | **Delta** | | | |
| --- | --- | --- | --- | --- | --- | --- | --- | --- | --- | --- | --- | --- | --- | --- | --- | --- | --- | --- | --- | --- |
|  | **B** | **4w** | **12w** | **24w** | **B** | **4w** | **12w** | **24w** | **B** | **4w** | **12w** | **24w** | **B** | **4w** | **12w** | **24w** | **B** | **4w** | **12w** | **24w** |
| 1 | 66.6 | 97.6 | 97.1 | 96.9 | 17.3 | 63.0 | 28.3 | 16.6 | 41.3 | 98.0 | 96.8 | 93.8 | 11.2 | 93.7 | 89.2 | 82.8 | 57.6 | 97.9 | 97.3 | 94.9 |
| 2 | 68.4 | 97.3 | 95.9 | 94.6 | 14.7 | 69.1 | 34.4 | 9.3 | 54.2 | 97.8 | 96.1 | 88.8 | 29.3 | 96.2 | 94.9 | 85.7 | 55.3 | 97.4 | 95.6 | 91.1 |
| 3 | 78.2 | 97.6 | 97.3 | 97.1 | 19.0 | 60.3 | 35.6 | 24.7 | 55.1 | 98.0 | 97.3 | 94.8 | 31.0 | 95.3 | 93.4 | 85.8 | 67.3 | 98.0 | 97.7 | 96.6 |
| 4 | 7.5 | 79.1 | 31.1 | 31.4 | 22.6 | -5.6 | 6.4 | 16.1 | 17.2 | 66.2 | 18.5 | 21.8 | -15.2 | 40.3 | 25.4 | 33.2 | 15.2 | 74.4 | 24.3 | 27.9 |
| 5 | 86.5 | 97.4 | 97.3 | 96.9 | -18.5 | 28.6 | -22.7 | -30.2 | 69.5 | 97.8 | 97.3 | 94.6 | 52.1 | 94.6 | 91.5 | 86.1 | 85.1 | 98.0 | 97.8 | 96.7 |
| 6 | 49.0 | 97.4 | 96.8 | 96.8 | 20.3 | 68.2 | 48.3 | 22.9 | 39.2 | 97.9 | 97.5 | 97.1 | 17.5 | 96.1 | 95.7 | 93.8 | 46.9 | 97.4 | 97.4 | 97.1 |
| 7 | 29.1 | 97.3 | 95.6 | 79.8 | 21.3 | 31.2 | 7.3 | 4.7 | 32.1 | 97.4 | 90.5 | 64.6 | 4.1 | 92.1 | 82.3 | 58.8 | 33.9 | 97.6 | 94.9 | 80.4 |
| 8 | 51.2 | 94.5 | 94.2 | 81.9 | 19.9 | 0.8 | 7.8 | -1.2 | 36.0 | 95.8 | 87.6 | 66.5 | 7.8 | 91.6 | 82.4 | 61.7 | 41.9 | 93.1 | 90.8 | 75.9 |
| 9 | 55.3 | 97.3 | 97.3 | 95.4 | 21.5 | 85.9 | 55.4 | 22.8 | 35.8 | 97.6 | 96.3 | 87.5 | 16.1 | 95.8 | 94.8 | 86.8 | 42.7 | 97.8 | 97.4 | 93.2 |
| 10 | 14.1 | 95.5 | 83.2 | 68.5 | 17.5 | 0.4 | -5.3 | -7.5 | 19.6 | 90.4 | 65.6 | 48.4 | -5.8 | 68.2 | 62.8 | 53.4 | 20.4 | 92.0 | 78.4 | 65.3 |
| 11 | 72.9 | 96.6 | 90.5 | 79.5 | 20.5 | 32.5 | 17.1 | 17.8 | 54.4 | 94.8 | 82.3 | 65.6 | -7.0 | 87.7 | 80.2 | 64.5 | 28.6 | 93.3 | 80.6 | 69.9 |
| 12 | 17.0 | 97.6 | 97.3 | 95.5 | 18.7 | 13.0 | -3.0 | 10.4 | 17.0 | 97.2 | 94.6 | 89.6 | -18.3 | 91.0 | 84.8 | 79.7 | 22.3 | 97.3 | 95.6 | 91.7 |
| 13 | 36.5 | 96.8 | 95.8 | 95.1 | 18.9 | 17.3 | 13.0 | 20.6 | 30.4 | 94.7 | 91.6 | 90.6 | 9.3 | 85.4 | 84.9 | 83.8 | 34.0 | 95.1 | 91.8 | 90.7 |
| 14 | 65.4 | 97.8 | 97.3 | 97.0 | 18.9 | 70.3 | 38.8 | 25.9 | 48.7 | 98.1 | 97.6 | 96.7 | 31.4 | 96.3 | 95.2 | 94.1 | 53.1 | 97.9 | 97.5 | 96.8 |
| 15 | 53.0 | 97.6 | 96.8 | 96.3 | 18.7 | -15.4 | -18.3 | -16.1 | 40.5 | 97.3 | 95.7 | 93.9 | 30.4 | 94.9 | 93.4 | 91.9 | 47.4 | 97.6 | 96.4 | 95.4 |
| 16 | 21.4 | 97.6 | 93.3 | 92.1 | 21.4 | -15.6 | -12.0 | -21.4 | 19.3 | 94.0 | 79.5 | 85.0 | -14.6 | 79.6 | 63.6 | 65.0 | 26.0 | 97.4 | 91.5 | 90.1 |
| 17 | 52.6 | 97.6 | 97.0 | 96.5 | 16.0 | 74.5 | 40.1 | 22.9 | 34.4 | 97.9 | 97.0 | 95.8 | 3.6 | 95.9 | 93.0 | 89.3 | 42.9 | 97.8 | 97.5 | 96.6 |
| 18 | 77.7 | 97.8 | 97.3 | 97.2 | 17.8 | 42.3 | 9.1 | 11.0 | 58.7 | 98.0 | 96.5 | 97.2 | 28.8 | 95.4 | 88.4 | 90.3 | 52.6 | 97.9 | 96.3 | 96.8 |
| 19 | 61.8 | 97.1 | * | * | 33.0 | 67.9 | * | * | 51.1 | 94.8 | * | * | 49.0 | 93.6 | * | * | 52.0 | 94.9 | * | * |
| 20 | 61.1 | 97.5 | 95.9 | 89.3 | 15.0 | 57.3 | 32.2 | 17.4 | 50.6 | 96.7 | 92.8 | 79.7 | 30.6 | 92.0 | 91.0 | 82.2 | 56.6 | 97.7 | 95.6 | 89.0 |
| 21 | 18.5 | 71.9 | 41.4 | 40.6 | 16.7 | -8.4 | 6.4 | 10.0 | 21.5 | 63.1 | 31.3 | 28.2 | 3.8 | 45.2 | 27.7 | 33.0 | 22.1 | 71.6 | 45.8 | 39.3 |
| 22 | 53.7 | 97.7 | 96.6 | 95.6 | 3.7 | 12.0 | -14.6 | -20.0 | 46.9 | 98.0 | 96.0 | 93.6 | 32.3 | 95.0 | 88.3 | 81.6 | 53.3 | 98.0 | 96.9 | 94.9 |

B = 2-dose CoronaVac (before boosting); 4w, 12w, 24w = 4 weeks, 12 weeks and 24 weeks after BNT162b2 boosting.

*The subject died from sepsis.
